# High Incidence of HPV-Associated Head and Neck Cancers in FA Deficient Mice Is Associated with E7’s Induction of DNA Damage through Its Inactivation of Pocket Proteins

**DOI:** 10.1371/journal.pone.0075056

**Published:** 2013-09-23

**Authors:** Jung Wook Park, Myeong-Kyun Shin, Henry C. Pitot, Paul F. Lambert

**Affiliations:** McArdle Laboratory for Cancer Research, University of Wisconsin School of Medicine and Public Health, Madison, Wisconsin, United States of America; The University of North Carolina at Chapel Hill, United States of America

## Abstract

Fanconi anemia (FA) patients are highly susceptible to solid tumors at multiple anatomical sites including head and neck region. A subset of head and neck cancers (HNCs) is associated with ‘high-risk’ HPVs, particularly HPV16. However, the correlation between HPV oncogenes and cancers in FA patients is still unclear. We previously learned that FA deficiency in mice predisposes HPV16 E7 transgenic mice to HNCs. To address HPV16 E6’s oncogenic potential under FA deficiency in HNCs, we utilized HPV16 E6-transgenic mice (*K14E6*) and HPV16 E6/E7-bi-transgenic mice (*K14E6E7*) on genetic backgrounds sufficient or deficient for one of the *fanc* genes, *fancD2* and monitored their susceptibility to HNCs. *K14E6* mice failed to develop tumor. However, E6 and *fancD2*-deficiency accelerated E7-driven tumor development in *K14E6E7* mice. The increased tumor incidence was more correlated with E7-driven DNA damage than proliferation. We also found that deficiency of pocket proteins, pRb, p107, and p130 that are well-established targets of E7, could recapitulate E7’s induction of DNA damage. Our findings support the hypothesis that E7 induces HPV-associated HNCs by promoting DNA damage through the inactivation of pocket proteins, which explains why a deficiency in DNA damage repair would increase susceptibility to E7-driven cancer. Our results further demonstrate the unexpected finding that FA deficiency does not predispose E6 transgenic mice to HNCs, indicating a specificity in the synergy between FA deficiency and HPV oncogenes in causing HNCs.

## Introduction

Fanconi Anemia (FA), which is a rare heterogeneous and recessive genetic disease, displayed developmental defects and abnormalities in hematopoietic stem cells such as bone marrow failure and acute myeloid leukemia, the major phenotypes displayed in early childhood of the patients. To date 15 cellular genes (*fanc* genes) have been identified for which homozygous mutations are associated with the FA disease. *fanc* gene products (Fanc) are composing a DNA repair pathway called FA pathway with several other cellular proteins to help repairing damaged DNA, specifically DNA interstrand cross-links (ICLs) damage. Eight of Fanc proteins (FancA, B, C, E, F, G, L, and M) interact each other and form a protein complex called the FA core complex. FancL in the complex, which has an *E3* ubiquitin ligase activity put mono-ubiquitin on FancD2/I in the response of the DNA damage [1]. In addition, stalled DNA replication forks in S-phase activates the FA pathway [2]. The mono-ubiquitinated FancD2/I are localized to sites of DNA damage where they interact with other proteins known to be involved in repairing the damaged DNA, such as BRCA1, FancD1/BRCA2, FancN/PALB2 and Rad51 leading to DNA repair [3,4]. Disruption of this FA pathway leads to increased sensitivity to DNA cross-linkers, chromosomal instability, spontaneous sister chromatid exchange, and cell cycle perturbations [5,6]. Recent studies showed that mono-ubiquitinated FancD2 recruits critical effector molecules such as the FAN1 nuclease (or SLX4) via their ubiquitin-binding domains [7,8]. Thus, mono-ubiquitination of FancD2 is critical for not only its chromatin localization but also acting as a scaffold for specific DNA repair factors. The FA pathway acts together with homologous recombination repair system (HR) to maintain genomic instability [9]. Depletion of FancD2 inhibits HR response, indicating that HR acts downstream of FancD2 [10]. Increased genomic instability is believed to contribute to the increased susceptibility of FA patients to cancer. Hematopoietic stem cell transplantation (HSCT) could be a solution to help the defects in hematopoietic cells. Even after successful HSCT, squamous cell carcinomas (SCCs) at head and neck regions become a high risk for the patients [11-15].

A subset of head and neck cancers (HNCs) (~25%), primarily amongst tumors from the oropharynx in the general population is positive for high-risk human papillomaviruses (HPVs), particularly HPV type-16 (HPV16) encoding the three oncogene, E5, E6, and E7 [16,17]. In particular FA patients from North America a high percentage (84%) of HNC arising at various sites in the head and neck region (i.e. not restricted to the oropharynx) were found to be positive for high-risk HPVs [18]. Consistent with a role of HPV in these cancers, mutations in p53, a tumor suppressor that is inactivated by HPV16 E6, were not found in HNCs from these patients. However, in patients from European countries, HNCs were HPV DNA negative and over 50% of these tumors had p53 mutations [18,19]. Given these conflicting clinical data, it remains unclear whether HPVs play a major role in HNCs arising in FA patients. Several studies point to a contribution of FA deficiency in HPV-associated disease at the molecular level. In tissue culture, HPV16 E7 expression was shown to stimulate transcription of *fancD2* [20] and activate the FA pathway [21]. In another study making use of organotypic cultures of human keratinocytes to recapitulate the HPV life cycle, HPV16 and HPV18-induced epithelial hyperplasia was increased in *fancA*-deficient cells or in cells knocked down in its expression; whereas, restoration of *fancA* expression attenuated this effect [22]. These studies indicate that there is an interplay between HPV and the FA pathway.

Our laboratory developed an animal model for HPV-associated HNCs using HPV16 oncogene transgenic mice when treated with a chemical carcinogen, 4NQO, which induces DNA adducts akin to those caused by tobacco-associated carcinogens synergizes with HPV16 oncogenes to induce HNC [23]. The cancers arising in our animal model share histopathological and molecular properties with HPV-positive HNCs in humans. The site at which tumors arise is less restrictive than in humans, presumably reflective of the pattern of HPV transgene expression throughout the mouse oral/upper GI epithelia. Between E6 and E7, HPV16 E7 has greater potential to cause HNC in animal models [23,24] with E6 contributing to increased incidence in combination with E7 [25]. The contribution of HPV16 E5 to HNC in this mouse model has not been fully evaluated but appears to be much less than that of E7 (Strati and Lambert, unpublished results).

We previously assessed the influence of FA pathway on the incidence of HNCs in mice expressing just the HPV16 E7 oncogene. Deficiency in the FA pathway highly increased HPV16 E7-driven tumor incidence [26]. However, in HPV-positive human cancers, E7 is always found to be co-expressed along with E6. Therefore we pursued studies described herein to address influences FA pathway in head and neck carcinogenesis in the context of mice expressing E6 alone or together with E7. Specifically, we generated *K14E6* and *K14E6E7* transgenic mice on *fancD2*-sufficient and -deficient backgrounds. We observed a significant increase in tumor incidence and severity of neoplastic disease in the *K14E6E7*/*FancD2* ^*-/-*^ mice compared to the *K14E6E7*/*FancD2* ^*+/+*^ mice; however, *fancD2*-deficiency did not increase either readout for tumorigenesis in the *K14E6* mice expressing E6 alone. Both HPV16 E6 and E7 caused an increase in the frequency of cells supporting DNA synthesis and this was augmented on the FA-deficient background. Indeed FA deficiency alone led to an increase in cell proliferation. But this induction of cell proliferation did not correlate with the influence of the FA pathway on tumorigenesis. On the other hand, frequency in the epithelia of the tongue and esophagus of γ-H2AX nuclear foci positive cells, a marker for DNA damage response, did correlate with tumor incidence. This marker was induced in the tissues that expressed the E7 oncogene; whereas, E6 did not induce expression of this marker of DNA damage response. In addition, two biomarkers, MCM7 and p16, used to distinguish HPV-positive HNCs from HPV-negative HNCs were found to be highly up-regulated in *K14E6E7* mice regardless of *fancD2* gene status, indicating that they could be useful for determining the HPV status of HNCs arising in FA patients. Finally, we observed that deficiency in expression of the pocket proteins, pRb, p107, and p130, which are established targets of E7, could recapitulate E7’s induction of DNA damage responses in head and neck epithelia. Our current study supports the hypothesis that E7 induces HNC by promoting DNA damage through the inactivation of pocket proteins. This data can explain why deficiency in the FA pathway increases susceptibility to E7-driven cancer.

## Materials and Methods

### Mice

*K14E6* transgenic *mice* [27] *and K14E7* transgenic mice [28] on the FVB genetic background, were crossed to *fancD2* knockout (*FancD2* ^*-/-*^) mice [29], on the 129S4 genetic background, to generate F_1_ mice, *K14E6/FancD2* ^*+/-*^ and *K14E7/FancD2* ^*+/-*^ (FVB/129S4 mixed background). All experimental mice (*NTG/FancD2* ^*+/+*^, *NTG/FancD2* ^-/-^, *K14E6/FancD2* ^*+/+*^, *K14E6/FancD2* ^*-/-*^, *K14E6E7/FancD2* ^*+/+*^, and *K14E6E7/FancD2* ^*-/-*^ mice) were males generated by intercrossing F_1_ mice. *K14Cre/Rb* ^*f/f*^, *Rb f* ^/f^/*p130* ^*-/-*^, and *K14Cre/Rb^f/f^/p130* ^*-/-*^ were generated on the same *FVB/129/C57* mixed genetic background as described previously [30]. *Rb f* ^/f^/*p107* ^*-/-*^ and *K14CreER/Rb^f/f^/p107* ^*-/-*^ were generated on the same *CD1/129/C57* mixed genetic background as described previously [30]. All mice were genotyped by PCR. Mice were injected intraperitoneally with 0.3 mL bromodeoxyuridine (BrdUrd; 12.5 mg/mL) 1 hour before euthanasia to measure the rate of DNA synthesis. Tongues and esophagi were harvested and processed as previously described [23]. Mice were housed in the Association for Assessment of Laboratory Animal Care-approved McArdle Laboratory Animal Care Unit. All procedures were carried out in accordance with our animal protocol approved by the University of Wisconsin Institutional Animal Care and Use Committee.

### 4-nitroquinoline-1-oxide (4NQO) induced head and neck carcinogenesis study and histologic analysis

The treatment and guidelines for histological analysis of tumors were previously described [26]. Briefly, mice were treated with 4-nitroquinoline-1-oxide (4NQO; 10 µg/ml) in their drinking water for 8 weeks and then held off treatment for an additional 16 weeks. At the end point or when mice became moribund, they were euthanized, overt tumors in tongue and esophagus were scored, and tissues were collected for histopathological analyses.

### Immunofluorescence

Immunofluorescence was performed as described previously [31]. Antibodies used included anti-p16 (1:50 in 5% nonfat milk/5% horse serum; M156, Santa Cruz Biotech.), anti-Mcm7 (1:200 in 5% horse serum; Neomarkers), anti-cytokeratin 14 conjugated to FITC (CK14; 1:100 in 5% horse serum; CBL 197F, Millipore), anti-bromodeoxyuridine (BrdUrd; 1:50 in 5% horse serum; Calbiochem), and anti-γ-H2AX (1:100 in 5% horse serum; Millipore)

### Quantification of BrdUrd positive nuclei and γ-H2AX foci positive cells

At least three mice of each genotype, *NTG/FancD2* ^*+/+*^, *NTG/FancD2* ^*-/-*^, *K14E6/FancD2* ^*+/+*^, *K14E6/FancD2* ^*-/-*^, *K14E6E7/FancD2* ^*+/+*^, and *K14E6E7/FancD2* ^*-/-*^ mice, were selected and ~8 to 10 frames (400x) of cells within the suprabasal (Cytokeratin 14 negative) and basal (Cytokeratin 14 positive) layers of tongue and esophagus epithelia were quantified for each mouse.

### Statistical analysis

One-sided Barnard’s exact test was used to determine the significance of differences in overt tumor incidence between each group of mice. Two-sided Wilcoxon rank sum test was used to determine the significance of differences in severity of overt lesion in the animal tongue and esophagus. To determine the significance for BrdUrd positive nuclei and γ-H2AX nuclear foci positive cells between each group of mice, a two-sided Wilcoxon rank-sum test was used.

## Results

### Disruption of the FA pathway significantly increases the incidence of HPV-oncogene associated tumors in E6/E7 bi-transgenic mice, but not in E6 transgenic mice

To address the role of E6 and deficiency of the FA pathway in head and neck carcinogenesis, HPV16 E6 transgenic (*K14E6*) and HPV16 E7 transgenic (*K14E7*) mice were crossed to *fancD2*-null mice to generate four different genotypes; *K14E6*/*FancD2* ^*+/+*^, *K14E6*/*FancD2* ^*-/-*^, *K14E6E7*/*FancD2* ^*+/+*^, and *K14E6E7*/*FancD2* ^*-/-*^ mice. Mice were treated with 4NQO, a water soluble chemical carcinogen that acts as a co-carcinogen in inducing HNCs in our HPV16 transgenic mice [23], for 8 weeks and then held for an additional 16 weeks. At the 24 week endpoint, tongues and esophagi were harvested, scored for overt tumors, and the tissues were fixed, paraffin-embedded, sectioned and subjected to histopathological analyses to assess the worst stage of neoplastic disease.

On *fancD2*-sufficient background, only two of 26 *K14E6/FancD2* ^*+/+*^ mice (8%) developed overt head and neck tumors, which was not significantly different from that observed in nontransgenic (NTG) mice (Table 1). This is consistent with our prior study [25]. On the *fancD2*-deficient background, two out of 22 *K14E6/FancD2* ^*-/-*^ mice (9%) developed overt tumors (Table 1), which was not significantly different from that observed on the *fancD2*-sufficient background (*K14E6/FancD2* ^*+/+*^ vs. *K14E6/FancD2* ^*-/-*^, *P*=0.52). Thus, *fancD2* deficiency did not lead to an increased susceptibility of E6 transgenic mice to head and neck tumors.

**Table 1:** Comparison of 4NQO induced overt tumor incidences in the mouse tongue and esophagus tissues.

***Genotype***	**Group size, n**	**Animal tissues with overt tumor, n (%**)		
		**Tongue & Esophagus**	**Tongue**	**Esophagus**
^*^NTG/FancD2^*+*^^*/*^^*+*^	24	0 (0)	0 (0)	0 (0)
^*^NTG/FancD2^*-/-*^	26	0 (0)	0 (0)	0 (0)
^*^K14E7/FancD2^*+*^^*/*^^*+*^	20	4 (20)	2 (10)	3 (15)
^*^K14E7/FancD2^*-/-*^	21	11 (52)	5 (24)	6 (29)
***K14E6/FancD2*^*+*^^*/*^^*+*^**	26	2 (8)	0 (0)	2 (8)
***K14E6/FancD2*^*-/-*^**	22	2 (9)	0 (0)	2 (9)
***K14E6E7/FancD2*^*+*^^*/*^^*+*^**	14	6 (43)	4^a^ (29)	4^a^ (29)
***K14E6E7/FancD2*^*-/-*^**	14	11 (79)	9^b^ (64)	7^b^ (50)

NOTE: *K14E6E7/FancD2* ^+ /^^+^ mice show a significant increase of tumor incidence comparing with *K14E6/FancD2* ^+ /^^+^ mice (*P* = 0.01). The difference in tumor incidences between *K14E6/FancD2* ^*+/+*^ and *K14E6/FancD2* ^*-/-*^ groups is showing no statistical significance (*P* = 0.52). However, the difference in the tumor incidences between *K14E6E7/FancD2* ^*+/+*^ and *K14E6E7/FancD2* ^*-/-*^ groups is statistically significant (*P* = 0.03). All statistical comparisons were performed using a one-sided Barnard’s exact test. ^a^ Two of the *K14E6E7/FancD2* ^*+/+*^ mice had tumors in the both tongue and esophagus. ^b^ Five of the *K14E6E7/FancD2* ^*-/-*^ mice had tumors in the both tongue and esophagus.

^*^ Overt tumor incidences of *NTG/FancD2* ^+ */*^ ^+^ *NTG/FancD2* ^- ^/- *K14E7/FancD2* ^*+/+*^, and *K14E7/FancD2* ^*-/-*^ mice are from our previous study [26]

We next looked at the influence of FA-deficiency on head and neck tumorigenesis in mice expressing both E6 and E7. On the *fancD2*-sufficient background, *K14E6E7/FancD2* ^*+/+*^ mice developed an increased number of overt head and neck tumors compared to *K14E7/FancD2* ^*+/+*^ mice (Table 1), consistent with our prior studies demonstrating an ability of E6 to augment the efficiency of E7-driven head and neck tumorigenesis [25]. On the *fancD2*-deficient background, we observed a significant increase in the incidence of overt tumors in *K14E6E7* mice, from 43% to 79% (*K14E6E7/FancD2* ^*+/+*^ vs. *K14E6E7/FancD2* ^*-/-*^, *P*=0.03 in Table 1). The magnitude of the increase in frequency of overt tumors on the *fancD2*-deficient background seen with the E6/E7 bi-transgenic mice was no greater than that seen with E7 only transgenic mice (Table 1). This data indicates that the FA pathway influences the oncogenic potential of E7 but not E6.

### Deficiency in FA pathway led to more aggressive neoplastic HPV-associated disease

We performed detailed histopathological analysis of the tissue harvested from these mice to assess the grade of neoplastic disease in these cohorts of mice. Tissues with overt tumors were harvested, fixed, paraffin-embedded and sections subjected to histopathology. Specifically, every tenth 5µm section of the tongue and esophagus was stained with hematoxylin and eosin (H&E) and analyzed to determine the worst stage of neoplastic disease in each animal (Table 2). HPV16 oncogene-transgenic mice develop a progressive disease, ranging from normal epithelium and benign papilloma to invasive cancer (i.e., carcinoma) [24,26]. Based on the level of differentiation (keratinization) within the carcinomas, cancers were sub-classified into grades I, II, and III. In the tongue, we only observed four benign papillomas in *K14E6E7*/*FancD2* ^*+/+*^ mice, whereas in the *K14E6E7*/*FancD2* ^*-/-*^ mice we observed three benign papillomas, four grade I carcinomas, and two grade II carcinomas. This increase in the severity of disease was statistically significant (*P*=0.02 in Table 2). While FA-deficiency also led to an increased severity of disease in the esophagi of mice with tumors, this increase was not statistically significant (*P*=0.25 in Table 2).

**Table 2:** Histopathological analysis of samples with overt lesions in the animal tongue and esophagus tissues.

**Tissue**	***Genotype***	**# of mice, n**	**Disease grade of overt lesion, n (%**)				
			**No disease**	**Papilloma**	**Grade of Carcinoma**		
					**I**	**II**	**III**
**Tongue**	***K14E6/FancD2*^*+/+*^**	26	26 (100)	-	-	-	-
	***K14E6/FancD2*^*-/-*^**	22	22 (100)	-	-	-	-
	***K14E6E7/FancD2*^*+/+*^**	14	10 (71)	4 (29)	-	-	-
	***K14E6E7/FancD2*^*-/-*^**	14	5 (36)	3 (21)	4 (29)	2 (14)	-
**Esophagus**	***K14E6/FancD2*^*+/+*^**	26	24 (92)	2 (8)	-	-	-
	***K14E6/FancD2*^*-/-*^**	22	20 (91)	2 (9)	-	-	-
	***K14E6E7/FancD2*^*+/+*^**	14	10 (71)	3 (21)	-	1 (7)	-
	***K14E6E7/FancD2*^*-/-*^**	14	7 (50)	5 (36)	-	2 (14)	-

NOTE: The overall disease grade in *K14E6E7/FancD2* ^*-/-*^ mice groups is statistically more severe than it in *K14E6E7/FancD2* ^*+/+*^ mice groups in the animal tongue tissue (*P* = 0.02), but not in the animal esophagus tissue (*P* = 0.25). All statistical analyses were performed by using a two-sided Wilcoxon rank-sum test.

### Two biomarkers, MCM7 and p16, are highly expressed in tumors in K14E6E7 mice regardless of fancD2 gene status

**Figure 1:**
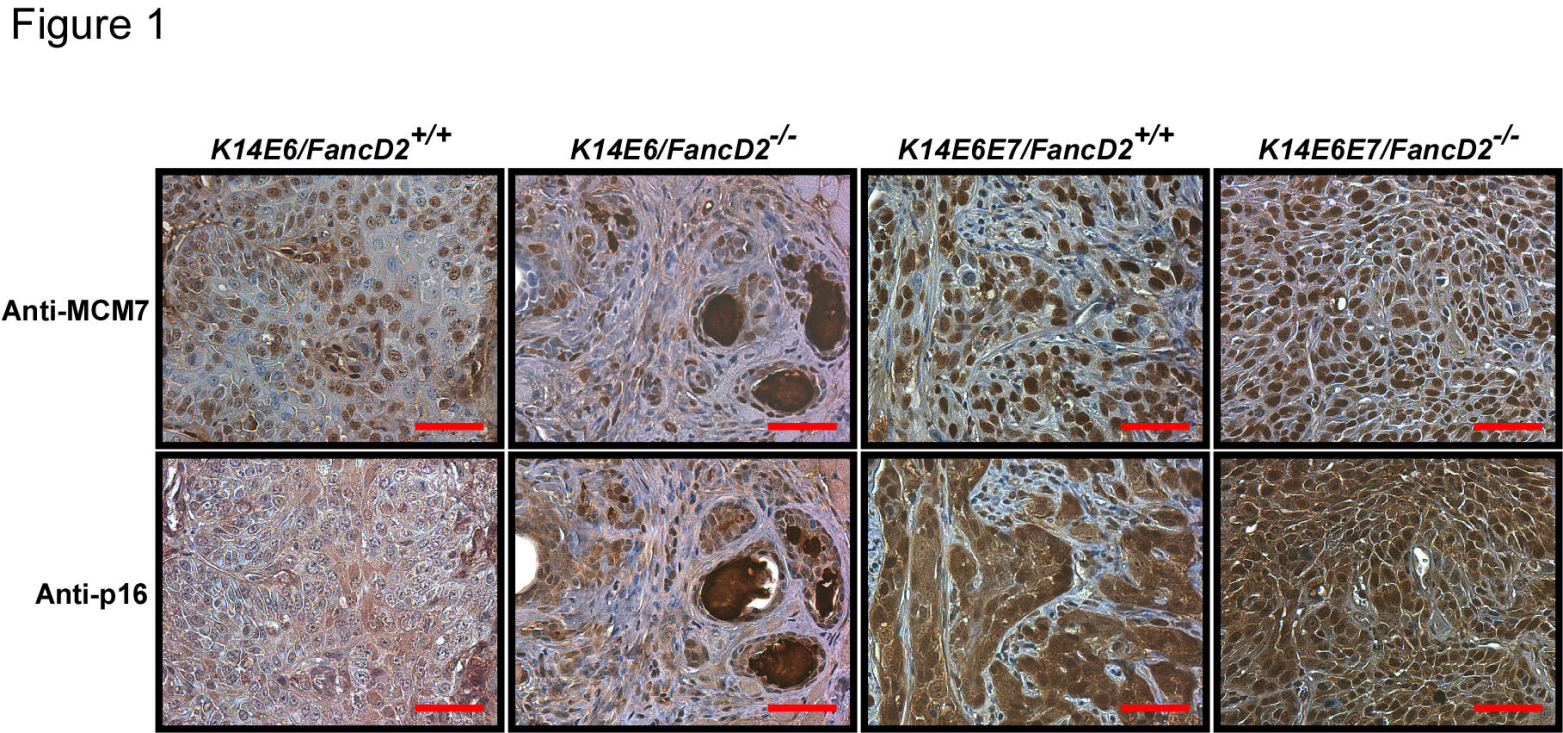
Expression of MCM7 and p16, two biomarkers in carcinomas arising on the tongue and esophagus in *K14E6* and *K14E6E7* mice on the *fancD2*-sufficient and -deficient backgrounds. Shown are representative immunohistochemistry images from sections stained with anti-MCM7 (brown nuclei) and anti-p16 (brown nuclei) antibodies and counterstained with hematoxylin (blue nuclei). Scale bar, 50 µm.

In the general population, HPV-positive HNCs are more responsive to radiation-therapy than HPV-negative HNCs [32]. However, this standard of care treatment for patients with HNCs is not applicable to FA patients because of the severe side effects of this therapy on these patients [33]. Identifying the HPV-status in HNCs from FA patients would help clinicians seek safer, and better solutions to the cure of these malignancies. Several previous studies including ours have demonstrated two molecular biomarkers, MCM7 and p16, are useful in distinguishing HPV-positive from HPV-negative HNCs both in mice [23,26] and in humans [34,35]. We wanted to monitor if HPV16 E6 and E7 double expression on the deficient FA pathway background also increases MCM7 and p16 protein levels in tumors from *K14E6E7*/*FancD2* ^*-/-*^ mice. Tissue sections from tumors arising in *K14E6E7*/*FancD2* ^*+/+*^ and *K14E6E7*/*FancD2* ^*-/-*^ mice were subjected to immunohistochemistry for anti-MCM7 and -p16 specific antibodies. No neoplastic lesions were observed in *NTG/FancD2* ^*+/+*^ and *NTG/FancD2* ^*-/-*^ mice. As previously reported [23], E6 did not increase the expression levels of these markers and *fancD2*-deficiency did not alter this finding (Figure 1). The two biomarkers were highly expressed in the nuclei of all of the benign and malignant tumors in *K14E6E7* mice regardless of *fancD2* expression status (Figure 1). Additionally, *fancD2* knockout did not change the expression pattern of these biomarkers in the normal epithelia of the tongue and esophagus (data not shown). These findings lead us to suggest that these two biomarkers will be valuable for diagnosing HPV-status in cancers from FA patients.

### Cell proliferation induced by HPV16 oncogenes is increased on the fancD2 deficient background

**Figure 2:**
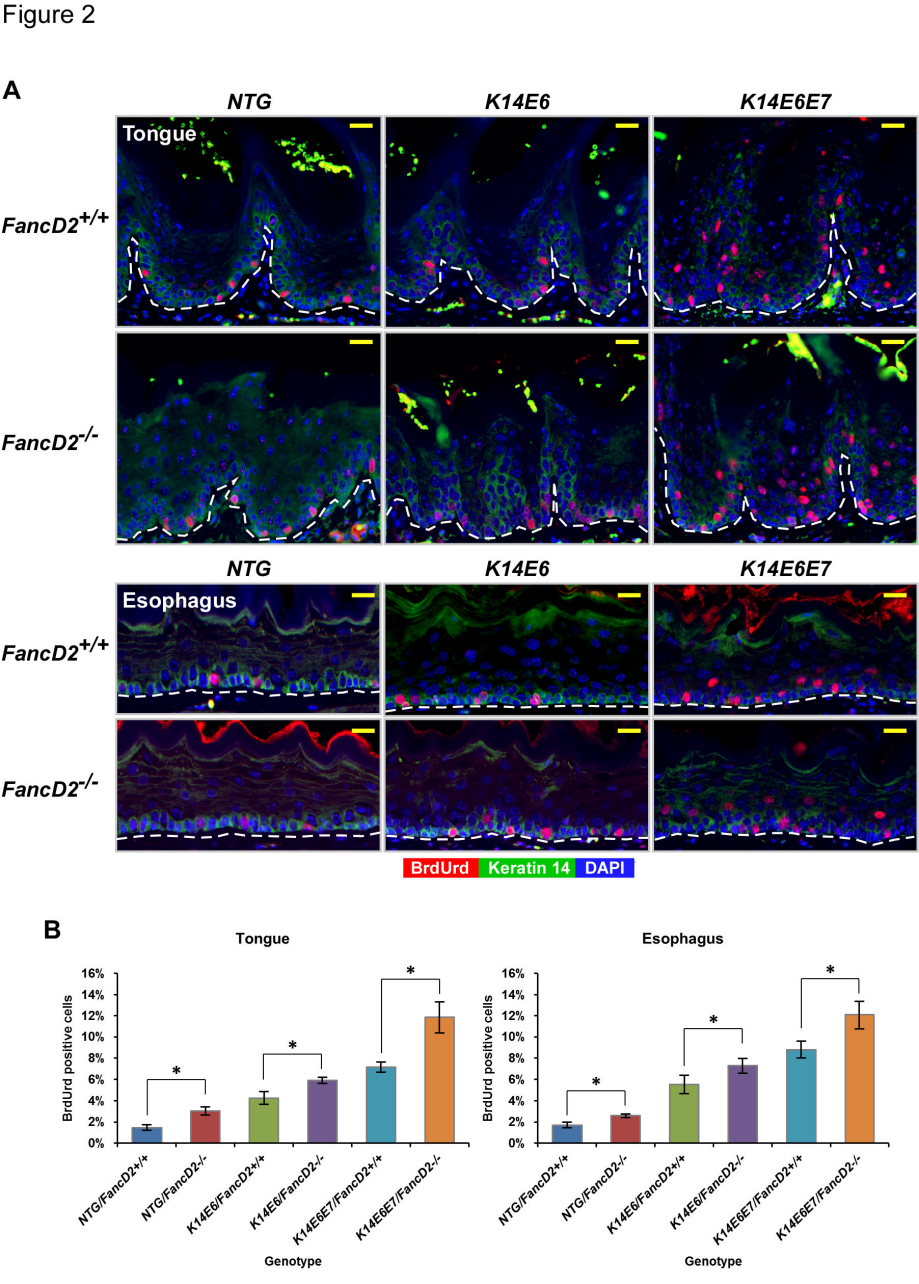
Examination of roles of HPV16 E6 and E7 on cell proliferation under the deficiency of *fancD2* gene. **A**, to score the newly synthesized DNA at the epithelial layers of the tongue and esophagus tissues, mice were intraperitoneally injected with BrdUrd before sacrifice and their tissues were stained for anti-BrdUrd (red) and anti-cytokeratin 14 (CK14) protein (green) antibodies. DAPI (blue) is used for a nuclear counterstaining. Scale bar, 20µm. **B**, at least three mice of each genotype, *NTG/FancD2* ^*+/+*^, *NTG/FancD2* ^*-/-*^, *K14E6/FancD2* ^*+/+*^, *K14E6/FancD2* ^*-/-*^, *K14E6E7/FancD2* ^*+/+*^, and *K14E6E7/FancD2* ^*-/-*^ mice, were selected and ~8 to 10 frames of cells at the epithelial layers of the tongue and esophagus epithelia were quantified for each mouse. The amount of BrdUrd-positive nuclei over the number of total cells was plotted in each case (columns); bars, SD. Asterisk (*) means that the deficiency of *fancD2* gene caused a significant increase in DNA synthesis between genotypes (*P*<0.05). All statistical comparisons were performed using a two-sided Wilcoxon rank-sum test.

Cell proliferation needs to be strictly regulated to maintain tissue homeostasis, and its deregulation contributes to neoplastic diseases. In our prior study we learned that HPV16 E7 and *fancD2*-deficiency additively and independently increased the frequency of cells in the basal epithelia of tongue and esophagus undergoing DNA synthesis [26]. An *in vitro* study demonstrated that FA deficiency led to hyperplasia in the context of organotypic raft cultures of human keratinocytes immortalized by the combination of E6 and E7 oncogenes [22]. To assess whether *in vivo* FA deficiency had any influence on cell proliferation in the presence of E6 alone or E6 and E7 together, the mice from the above head and neck cancer study were intraperitoneally injected with BrdUrd (12.5mg/ml), a synthetic thymidine analogue, 1 hour prior to sacrifice and BrdUrd-specific immunofluorescence was performed to identify cells supporting DNA synthesis in the epithelia lining the tongue and esophagus of the mice (Figure 2A). The frequency of BrdUrd-positive cells was quantified to provide a proliferative index for each tissue (Figure 2B).

On the *fancD2*-sufficient background, E6 alone or together with E7 significantly increased the number of BrdUrd-positive cells in the epithelium of the tongue or esophagus (*NTG/FancD2* ^*+/+*^ vs. *K14E6/FancD2* ^*+/+*^ or *K14E6E7/FancD2* ^*+/+*^, *P*<0.05 in Figure 2B). The combination of E6 and E7 led to a significantly increased number of BrdUrd-positive epithelial cells on *fancD2*-sufficient background than did E6 alone (*P*<0.05 in Figure 2B). Importantly, the frequency of cells supporting DNA synthesis was significantly increased in the epithelial cells of tongue or esophagus of all mice that were deficient for *fancD2*, regardless of the HPV16 oncogene status (*NTG/FancD2* ^*+/+*^ vs. *NTG/FancD2* ^*-/-*^, *K14E6*/*FancD2* ^*+/+*^ vs. *K14E6*/*FancD2* ^*-/-*^, and *K14E6E7*/*FancD2* ^*+/+*^ vs. *K14E6E7*/*FancD2* ^*-/-*^; all *P*<0.05 in Figure 2B). We further refined our analysis of the influence of HPV16 oncogenes and FA deficiency on the induction of DNA synthesis to two different sub-compartments of the epithelia: the basal compartment, which is cytokeratin 14 (CK14) positive, and the suprabasal compartment, which is CK14 negative (Figure 2A). The above stated influence of both factors (HPV16 oncogenes and FA-status) was more uniformly observed in the basal compartment of both the tongue and esophagus (Figure S1). In summary, deficiency in *fancD2* led to the induction of cellular DNA synthesis in the tongue and esophagus epithelia regardless of HPV16 oncogene status. These results support the hypothesis that FancD2 plays a role of regulating S-phase cell cycle [6,36,37]. However, the fact that FA-deficiency did not cause an increased susceptibility to head and neck tumorigenesis in the E6 transgenic mice but did cause an increase in cell proliferation in these same mice indicates that induction of epithelial hyperplasia does not explain how FA deficiency drives tumorigenesis.

### The FA pathway specifically suppress E7-driven DNA damage

**Figure 3:**
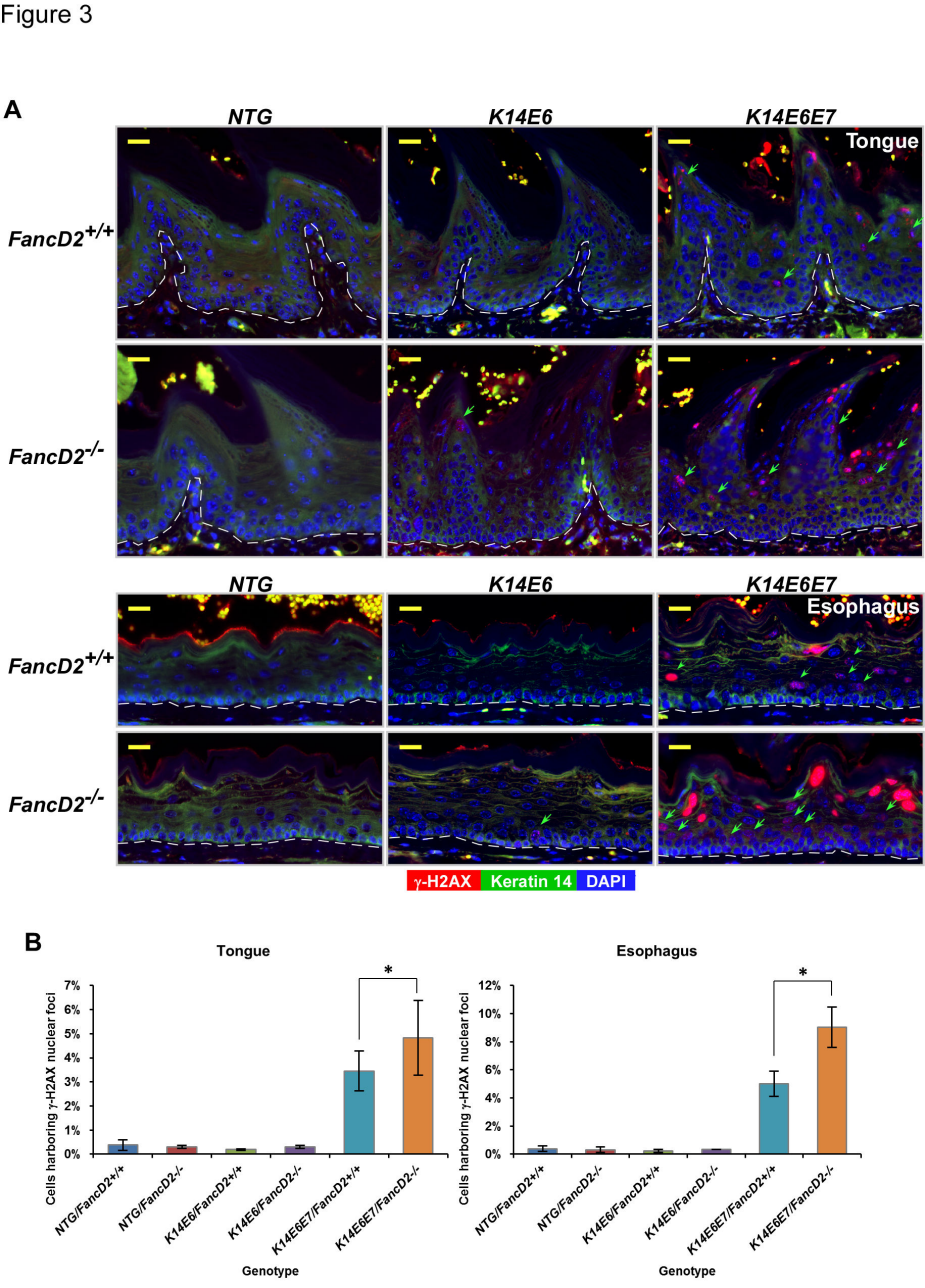
HPV16 E7-dependent DNA damage response via γ-H2AX was accelerated by the deficiency of *fancD2* gene. **A**. To assess induced DNA damage response by HPV16 E6 and E7 in the deficiency of *fancD2* gene, tissues from each group were stained for anti-γ-H2AX (red) and anti-cytokeratin 14 (CK14) protein (green) antibodies. DAPI is used for a nuclear counterstaining. Scale bar, 20µm. γ-H2AX foci positive cells are highlighted by green arrows in the images. **B**. At least three mice of each genotype, *NTG/FancD2* ^*+/+*^ *, NTG/FancD2* ^*-/-*^, *K14E6/FancD2* ^*+/+*^, *K14E6/FancD2* ^*-/-*^, *K14E6E7/FancD2* ^*+/+*^, and *K14E6E7/FancD2* ^*-/-*^ mice, were selected and ~8 to 10 frames of cells at the epithelial layers of the tongue and esophagus were quantified for each mouse. The amount of cells with γ-H2AX positive foci cells over the number of total cells was plotted in each case (columns); bars, SD. Asterisk (*) means that the differences in the number of cells with γ-H2AX foci between the groups were statistically compared (*P*<0.05). All statistical comparisons were performed using a two-sided Wilcoxon rank-sum test. Note: Exposure times used were the same for all samples analyzed. Some cells in tissues of *K14E6E7/FancD2* ^*+/+*^ and *K14E6E7/FancD2* ^*-/-*^ mice had very bright γ-H2AX-specific nuclear staining at the exposure used. Shorter exposure confirmed that these cells retained punctuate nuclear staining reflective of DNA damage response foci.

Both HPV16 E6 and E7 oncogenes have been argued to induce DNA damage based upon comet assays performed on cells in tissue culture [38,39]. Given that the FA pathway is involved in repair of damaged DNA, it would stand to reason that the level of DNA damage caused by E6 and E7 would be increased on an FA deficient background. The presence of DNA damage in cells can be indirectly scored by monitoring the presence of nuclear foci positive for histone 2AX phosphorylated at serine-139 (γ-H2AX) [40,41]. We had previously shown that in *K14E7* mice, E7 led to a robust increase in the frequency of γ-H2AX nuclear foci-positive cells in the epithelia of the tongue and esophagus, and the frequency was further increased on the *fancD2*-deficient background [26]. In the current study, we wanted to determine the role of HPV16 E6 on DNA damage response on the *fancD2*-sufficient and -deficient backgrounds. To this end, immunofluorescence staining specific for γ-H2AX was performed on sections of the tongue and esophagus from *NTG*, *K14E6*, and *K14E6E7* mice on the *fancD2-*sufficient or *fancD2*-deficient backgrounds (Figure 3A).

In the epithelium of tongue and esophagus tissue, E6 transgenic mice showed very few γ-H2AX foci positive cells, similar to that seen in the *NTG* mice. E6/E7 bi-transgenic mice, however, displayed a significantly increased frequency of γ-H2AX nuclear foci-positive cells (Figure 3B), indicating that E7 is necessary to induce DNA damage in the epithelial cells. *fancD2*-deficiency further increased the frequency of γ-H2AX foci positive cells in the tongue and esophagus of *K14E6E7* mice (*K14E6E7/FancD2* ^*+/+*^ vs. *K14E6E7/FancD2* ^*-/-*^; *P*<0.05 in Figure 3B) but no such increase was observed in the tissue from the *NTG* or *K14E6* mice. The increase in γ-H2AX nuclear foci-positive cells seen in the *K14E6E7* mice on either the *fancD2*-sufficient or *fancD2*-deficient backgrounds was primarily evident in the suprabasal epithelial compartment (Figure S2). These data indicate that HPV16 E7 is necessary for the induction of γ-H2AX nuclear foci-positive cells *in vivo*; whereas, HPV16 E6 is not sufficient. Importantly, the induction of this marker for DNA damage response correlated with the influence of FA-deficiency on tumorigenesis in the head and neck region.

### Deficiency of pocket protein family members, pRb and p130, is sufficient to account for E7’s driven DNA damage

**Figure 4:**
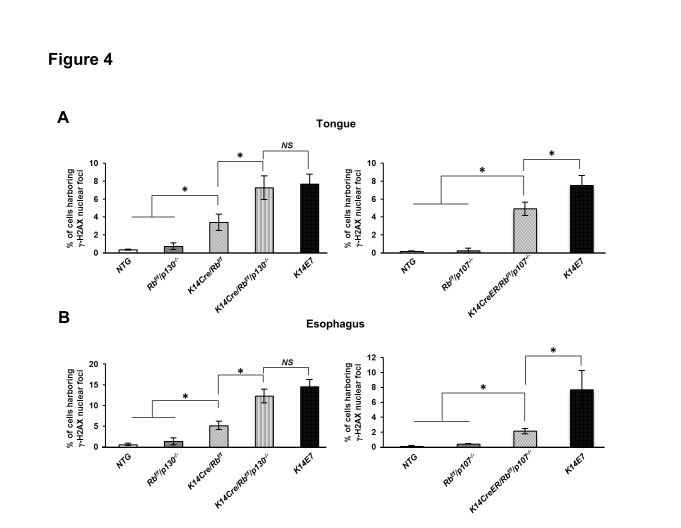
Deficiency of Pocket protein family members increased DNA damage via γ-H2AX in the tongue and esophagus epithelia. **A** and **B**. At least three mice from each genotype were randomly selected and more than eight image frames of cells at the epithelia of tongue and esophagus were quantified for each mouse. The amount of γ-H2AX nuclear-foci positive cells over total number of cells was plotted in each case (columns); bar, Standard deviation (SD). **A**. In the epithelial layer of the tongue tissue, *Rb f* ^/f^/*p130* ^*-/-*^ and *Rb f* ^/f^/*p107* ^*-/-*^ mice failed to increase the number of γ-H2AX nuclear-foci positive cells compared with *NTG* mice (*P*>0.05). *K14Cre/Rb* ^*f/f*^, *K14Cre/Rb^f/f^/p130* ^*-/-*^, and *K14CreER/Rb^f/f^/p107* ^*-/-*^ mice showed an increase in DNA damage (*P*<0.05). **B**. In the epithelial layer of the esophagus tissue, *Rb f* ^/f^/*p130* ^*-/-*^ and *Rb f* ^/f^/*p107* ^*-/-*^ mice failed to increase the number of γ-H2AX nuclear-foci positive cells compared with *NTG* mice (*P*>0.05). *K14Cre/Rb* ^*f/f*^, *K14Cre/Rb^f/f^/p130* ^*-/-*^, and *K14CreER/Rb^f/f^/p107* ^*-/-*^ mice showed a significant increase in DNA damage (*P*<0.05). Asterisk (*) means significant difference (*P*<0.05). *NS* means no significant difference (*P*>0.05). All statistical comparisons were performed using a two-sided Wilcoxon Rank sum test.

The observed induction in the formation of γ-H2AX nuclear foci, a marker for DNA damage responses, in the epithelia of tongue and esophagus tissues of E6/E7 bi-transgenic mice (Figure 3) is consistent with our prior observation that E7 alone is sufficient to induce DNA damage responses in the context of E7 transgenic mice [26]. This led us to question how E7 is driving the induction of cellular DNA damage responses. Mass-spectrometry based studies have documented that HPV16 E7 associates with more than a hundred different cellular proteins [42]. E7 can deregulate multiple cellular processes such as proliferation, apoptosis, and DNA damage response [43]. pRb is the most well-known protein of E7’s targets and pRb pocket family proteins, p107 and p130, are also considered relevant targets of E7 in the context of HPV-associated carcinogenesis [44]. To address if single or combined loss of these three pocket proteins recapitulates the observed E7-driven DNA damage in the epithelium of tongue and esophagus tissues, we utilized our germ-line or conditional knockout mice in pRb, p107, or p130 protein for avoiding or decreasing morbidity of mice [45]; *K14Cre/Rb* ^*f/f*^, *Rb f* ^/f^/*p130* ^*-/-*^, *K14Cre/Rb f* ^/f^/*p130* ^*-/-*^, *Rb f* ^/f^/*p107* ^*-/-*^, and *K14CreER/Rb^f/f^/p107* ^*-/-*^ mice. These mice were treated with 4-NQO for 8 weeks at 6-7 weeks of age. Immunofluorescence for anti-γ-H2AX was performed on sections of the tongue and esophagus from each genotype of mice. We found that in the epithelium of tongue or esophagus, deficiency in pRb significantly increased the frequency of cells harboring γ-H2AX nuclear foci compared with *NTG* mice (*NTG* vs. *K14Cre/Rb* ^*f/f*^, *P*<0.05 in Figure 4). However, p107 or p130 deficiency failed to increase the number of γ-H2AX nuclear foci positive cells (Figure 4). Double knockout of pRb/p107 or pRb/p130 caused a significant increase in γ-H2AX nuclear foci-positive cells *NTG* mice (*K14Cre/Rb^f/f^/p130* ^*-/-*^ or *K14CreER/Rb^f/f^/p107* ^*-/-*^ vs. *NTG*, *P*<0.05 in Figure 4). Loss of p130 in *K14Cre/Rb^f/f^/p130* ^*-/-*^ mice further increased the frequency of γ-H2AX nuclear foci-positive cells compared to mice deficient in pRb alone (*K14Cre/Rb* ^*f/f*^ vs. *K14Cre/Rb f* ^/f^/*p130* ^*-/-*^, *P*<0.05 in Figure 4). Interestingly, the induction in DNA damage response in pRb/p130 double knockout mice reached the same level as that observed in *K14E7* mice (*K14Cre/Rb^f/f^/p130* ^*-/-*^ vs. *K14E7*, *P*>0.05 in Figure 4). However, combined deficiency of pRb/p107 failed to reach this same level (*K14CreER/Rb^f/f^/p107* ^*-/-*^ vs. *K14E7*, *P*<0.05 in Figure 4). The increases in γ-H2AX nuclear foci-positive cells seen in those knockout mice were primarily evident in the suprabasal epithelial compartment of tongue or esophagus (Figure S3).

Taken together, these findings support the hypothesis that E7’s inactivation of pocket proteins, specifically pRb and p130, account for nearly all of E7’s capacity to induce DNA damage responses in head and neck epithelium of the mouse.

## Discussion

Whether high-risk HPV infection a caustic factor for the high incidence of squamous cell carcinomas in FA patients is an important question. In this study we provide experimental evidence that FA deficiency increases the susceptibility of mice expressing both the HPV16 E6 and E7 oncogenes to the development of head and neck cancer, but not in mice just expressing E6. This is an important finding as both E6 and E7 are commonly expressed in HPV-positive HNCs. Our data provide further support to the hypothesis that FA patients are increased in their susceptibility to HPV-induced HNCs.

### Deficiency in the FA pathway augments E7 but not E6 driven oncogenesis

We were surprised to find that FA deficiency failed to increase the susceptibility of E6 transgenic mice to HNCs; the influence of FA deficiency was specific to E7. Furthermore, in the presence of E7, E6 did not amplify the magnitude of the effect of FA deficiency on tumorigenesis. Thus, the influence of defects in the FA pathway on HPV-mediated oncogenesis is specific to the E7 oncogene. Both HPV16 E6 and E7 oncogenes have been reported to cause DNA damage based upon comet assays performed on viral oncogene-transduced cells grown in tissue culture [38]. We predicted that defects in the FA pathway would exacerbate the amount of damaged DNA accumulating in cells expressing either E6 or E7, and that this would lead to a heightened frequency of cells with γ-H2AX nuclear foci, a marker for DNA damage. However, this is not what we found. Only E7 led to an induction in the frequency of γ-H2AX nuclear foci-positive cells, which was further increased on the *fancD2*-deficient background. This raises the question: is the DNA damage scored for by the comet assay in E6 and in E7 expressing cells physiologically relevant? The comet assay used in the prior study was an alkaline comet assay that scores for both single strand and double strand DNA breaks, whereas the presence of γ-H2AX nuclear foci is thought to be a marker primarily of double strand DNA breaks. Both in our current *in vivo* study (Figure 4) and in prior tissue culture-based assays [38], E6 failed to induce formation of γ-H2AX nuclear foci. One possible explanation then for the specificity of the effect of FA deficiency on E7-driven carcinogenesis is that only E7 leads to an accumulation of double strand DNA breaks that are repaired in an FA pathway-dependent manner.

### Pocket proteins as relevant E7 targets in the induction of DNA damage

We found that double knockout of pRb and either p130 or p107, all of which are targets of inactivation by E7, increased DNA damage in our animal model (Figure 3). These findings support the hypothesis that endogenous DNA damage, which should be repaired before cells enter and/or pass through S phase, accumulates in cells not expressing pocket protein family members [46]. Our findings that DNA damage was much greater in mice deficient for two pocket proteins as opposed to any one pocket protein are also consistent with prior studies showing p107 and p130 are not simply replacements of pRb; rather, they possess specific tumor suppressing capabilities within different tissue contexts [47-50]. Furthermore, the increased levels of DNA damage caused by deletion of multiple pocket protein family members is consistent with our previous findings that combined deficiency of two pocket protein family members induced HNCs to a greater degree than mice deficient in any one pocket protein [30]. We conclude that E7’s ability to target multiple pocket protein family members contributes to its ability to induce DNA damage and accounts to a significant extent for HPV-associated head and neck carcinogenesis. Still, addressing the role of other E7’s target proteins in regulating DNA damage via γ-H2AX nuclear foci formation is worthy to understanding E7’s full oncogenic potential.

### Comparison of findings made here in the context of head and neck cancer to those made in the context of squamous carcinomas of the female lower reproductive tract

The surprising finding made here that FA deficiency does not increase the susceptibility of HPV16 E6 transgenic mice to HNCs whereas it does for mice carrying the HPV16 E7 oncogene led us to ask if the same difference is seen in another site of HPV-associated cancers, the female lower reproductive tract. Our rationale for pursuing these additional studies is that E6 appears to rely upon different activities to promote carcinogenesis in the two anatomical sites. This is evidenced by the observations that two mutant forms of HPV16 E6 reduced in either E6’s interaction with host proteins containing leucine rich domains (e.g. E6AP and E6BP1) or PDZ domains (e.g. Dlg and Scrib) are attenuated in their ability to drive cervical carcinogenesis [51], but not head and neck carcinogenesis [25]. Despite this difference, FA deficiency promoted E7- but not E6-driven carcinogenesis in the female lower reproductive tract (Park, in press) identical to what we observed here for the head and neck. Thus, the selective synergy between FA deficiency and E7-driven carcinogenesis is not tissue specific.

### Hit and run strategy of HPV in FA associated carcinogenesis

A very recent finding indicates the absence of HPV genomes in HNCs arising in FA patients [52]. This study is sequent to two earlier studies, one of which found HPV in FA-derived HNCs [18], the other of which did not [19]. If HPV is causally associated with HNCs in FA patients, could the virus be contributing in a hit and run mechanism? This possibility might be plausible were the defect in DNA repair in FA patients to lead to an accumulation of mutations that render the cancer no longer dependent upon viral oncogenes. We can now test this hypothesis using mice in which E7, the main viral oncogene driving HNC [24], can be regulated in its temporal expression [25].

## Supporting Information

Figure S1 **Examination of roles of HPV16 E6 and E7 on cell proliferation under the deficiency of *fancD2* gene.** At least three mice of each genotype, *NTG/FancD2* ^+/+^, *NTG/FancD2* ^-/-^, *K14E6/FancD2* ^+/+^, *K14E6/FancD2* ^-/-^, *K14E6E7/FancD2* ^*+/+*^, and *K14E6E7/FancD2* ^*-/-*^ mice, were selected and ~8 to 10 frames of cells at the suprabasal (CK14 negative) and basal (CK14 positive) layers of the tongue and esophagus epithelia were quantified for each mouse. The amount of BrdUrd-positive nuclei over the number of total cells was plotted in each case (columns); bars, SD. Asterisk (*) means that the deficiency of *fancD2* gene caused a significant increase in DNA synthesis between genotypes (*P*<0.05). All statistical comparisons were performed using a two-sided Wilcoxon rank-sum test.
(TIF)

Figure S2 **DNA damage response induced by HPV16 E7 via γ-H2AX under the deficiency of *fancD2* gene.** At least three mice of each genotype, *NTG/FancD2* ^*+/+*^ *, NTG/FancD2* ^*-/-*^, *K14E6/FancD2* ^*+/+*^, *K14E6/FancD2* ^*-/-*^, *K14E6E7/FancD2* ^*+/+*^, and *K14E6E7/FancD2* ^*-/-*^ mice, were selected and ~8 to 10 frames of cells at the basal (CK14 positive) and suprabasal (CK14 negative) layers of the tongue and esophagus epithelia were quantified for each mouse. The amount of cells with γ-H2AX positive foci cells over the number of total cells was plotted in each case (columns); bars, SD. Asterisk (*) means that the differences in the number of suprabasal epithelial cells with γ-H2AX foci between the groups were statistically compared (*K14E6E7/FancD2* ^*+/+*^ vs. *K14E6E7/FancD2* ^*-/-*^ *, P*=0.04/*P*=0.01 at tongue/esophagus). *NS* means no statistical difference. All statistical comparisons were performed using a two-sided Wilcoxon rank-sum test.
(TIF)

Figure S3 **Deficiency of Pocket protein family members Increased DNA damage via γ-H2AX in the tongue and esophagus epithelia.** A and B. At least three mice from each genotype were randomly selected and more than eight image frames of cells at the basal (CK14 positive) and suprabasal (CK14 negative) layers of the tongue (A) and esophagus (B) epithelia were quantified for each mouse. The amount of γ-H2AX nuclear-foci positive cells over total number of cells was plotted in each case (columns); bar, Standard deviation (SD). Asterisk (*) means significant difference (*P*<0.05). *NS* means no significant difference (*P*>0.05). All statistical comparisons were performed using a two-sided Wilcoxon Rank sum test.
(TIF)
