# Supplementary figures and images for: High Incidence of HPV-Associated Head and Neck Cancers in FA Deficient Mice Is Associated with E7’s Induction of DNA Damage through Its Inactivation of Pocket Proteins
Source: PLoS One. 2013 Sep 23;8(9):e75056. doi: 10.1371/journal.pone.0075056 (PMC3781031; doi:10.1371/journal.pone.0075056)

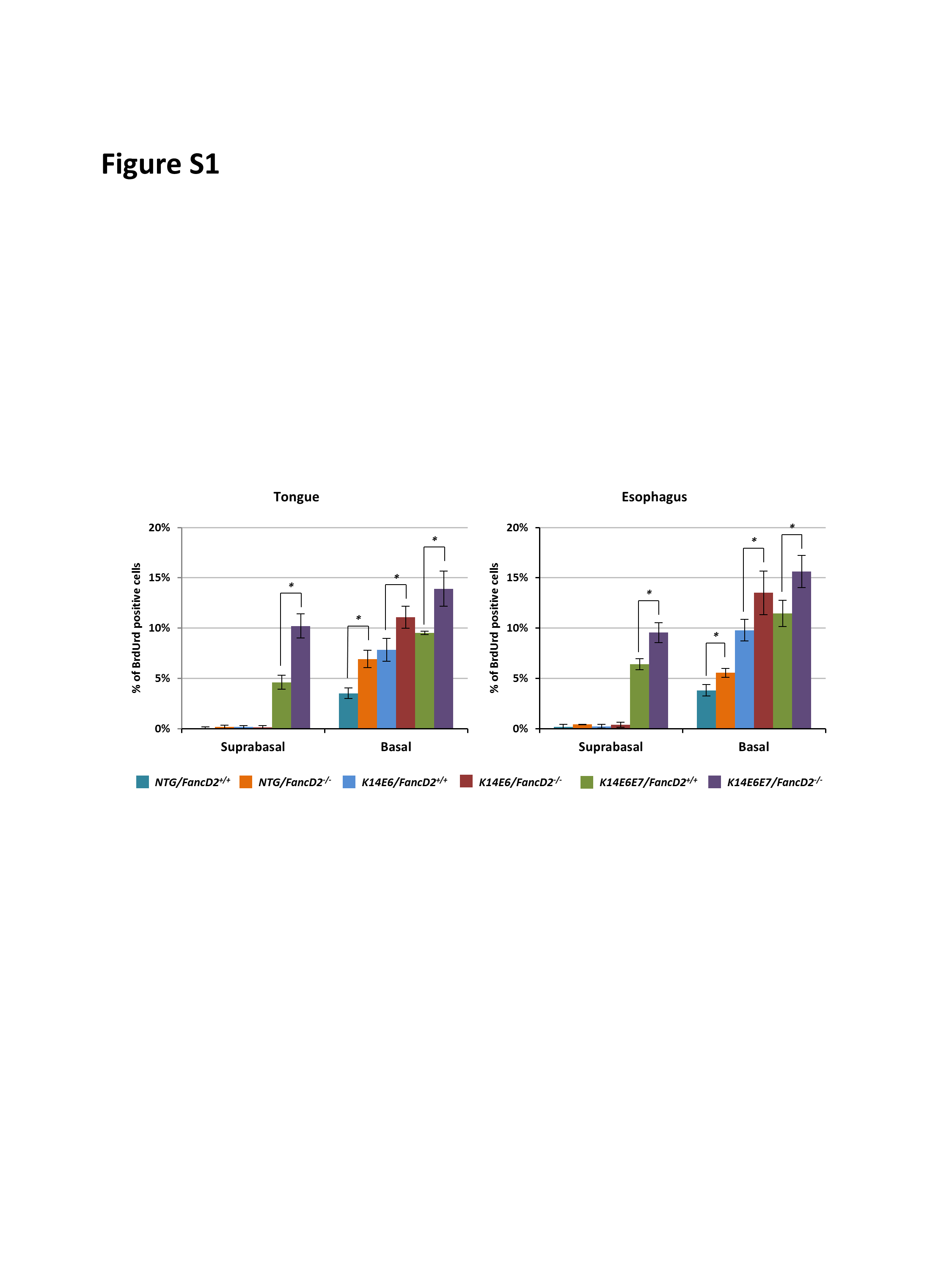

Supplement: Figure S1 — Examination of roles of HPV16 E6 and E7 on cell proliferation under the deficiency of fancD2 gene. At least three mice of each genotype, NTG/FancD2 +/+, NTG/FancD2 -/-, K14E6/FancD2 +/+, K14E6/FancD2 -/-, K14E6E7/FancD2 +/+, and K14E6E7/FancD2 -/- mice, were selected and ~8 to 10 frames of cells at the suprabasal (CK14 negative) and basal (CK14 positive) layers of the tongue and esophagus epithelia were quantified for each mouse. The amount of BrdUrd-positive nuclei over the number of total cells was plotted in each case (columns); bars, SD. Asterisk (*) means that the deficiency of fancD2 gene caused a significant increase in DNA synthesis between genotypes (P<0.05). All statistical comparisons were performed using a two-sided Wilcoxon rank-sum test. (TIF) [file pone.0075056.s001.tif]

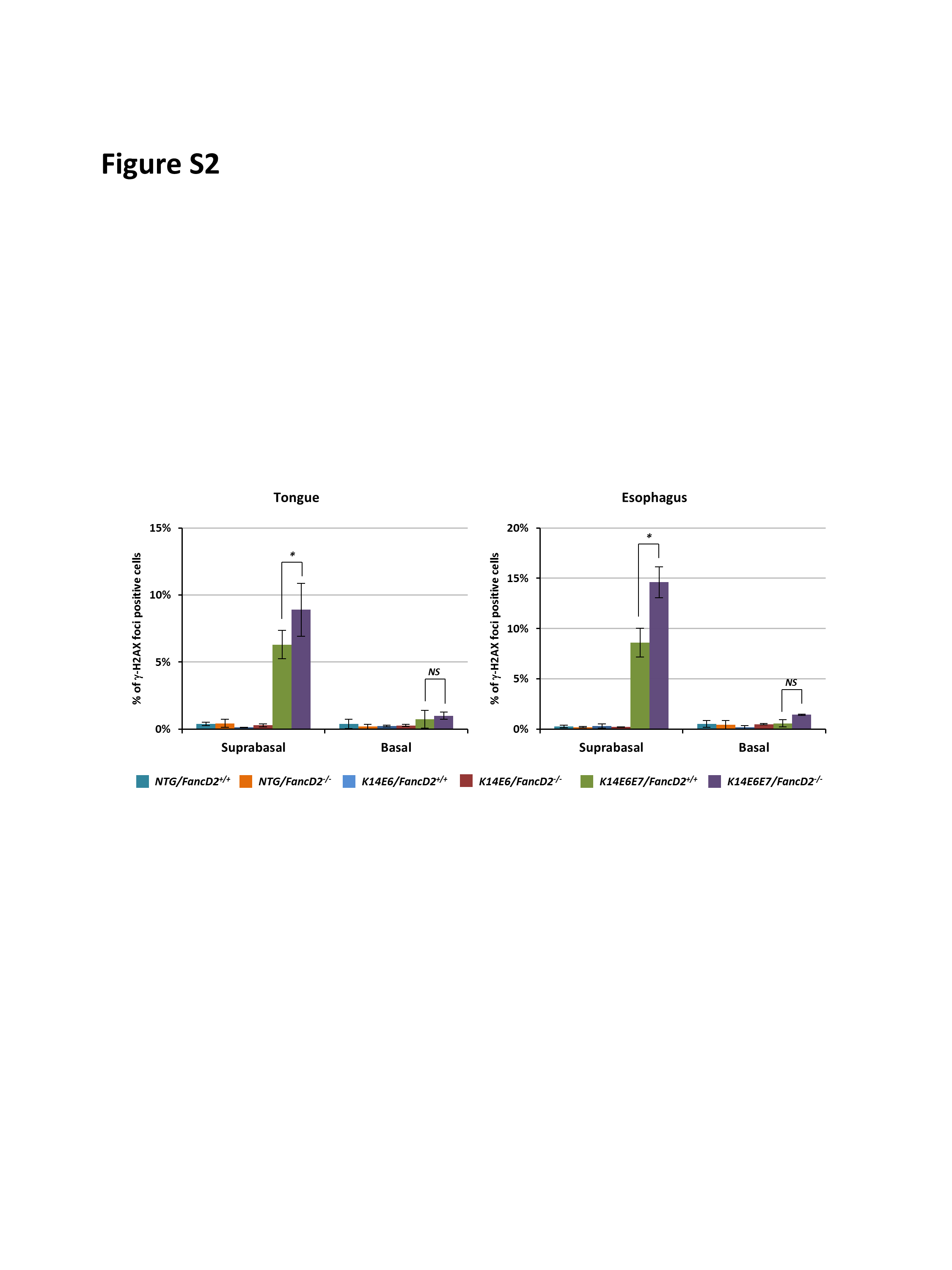

Supplement: Figure S2 — DNA damage response induced by HPV16 E7 via γ-H2AX under the deficiency of fancD2 gene. At least three mice of each genotype, NTG/FancD2 +/+ , NTG/FancD2 -/-, K14E6/FancD2 +/+, K14E6/FancD2 -/-, K14E6E7/FancD2 +/+, and K14E6E7/FancD2 -/- mice, were selected and ~8 to 10 frames of cells at the basal (CK14 positive) and suprabasal (CK14 negative) layers of the tongue and esophagus epithelia were quantified for each mouse. The amount of cells with γ-H2AX positive foci cells over the number of total cells was plotted in each case (columns); bars, SD. Asterisk (*) means that the differences in the number of suprabasal epithelial cells with γ-H2AX foci between the groups were statistically compared (K14E6E7/FancD2 +/+ vs. K14E6E7/FancD2 -/- , P=0.04/P=0.01 at tongue/esophagus). NS means no statistical difference. All statistical comparisons were performed using a two-sided Wilcoxon rank-sum test. (TIF) [file pone.0075056.s002.tif]

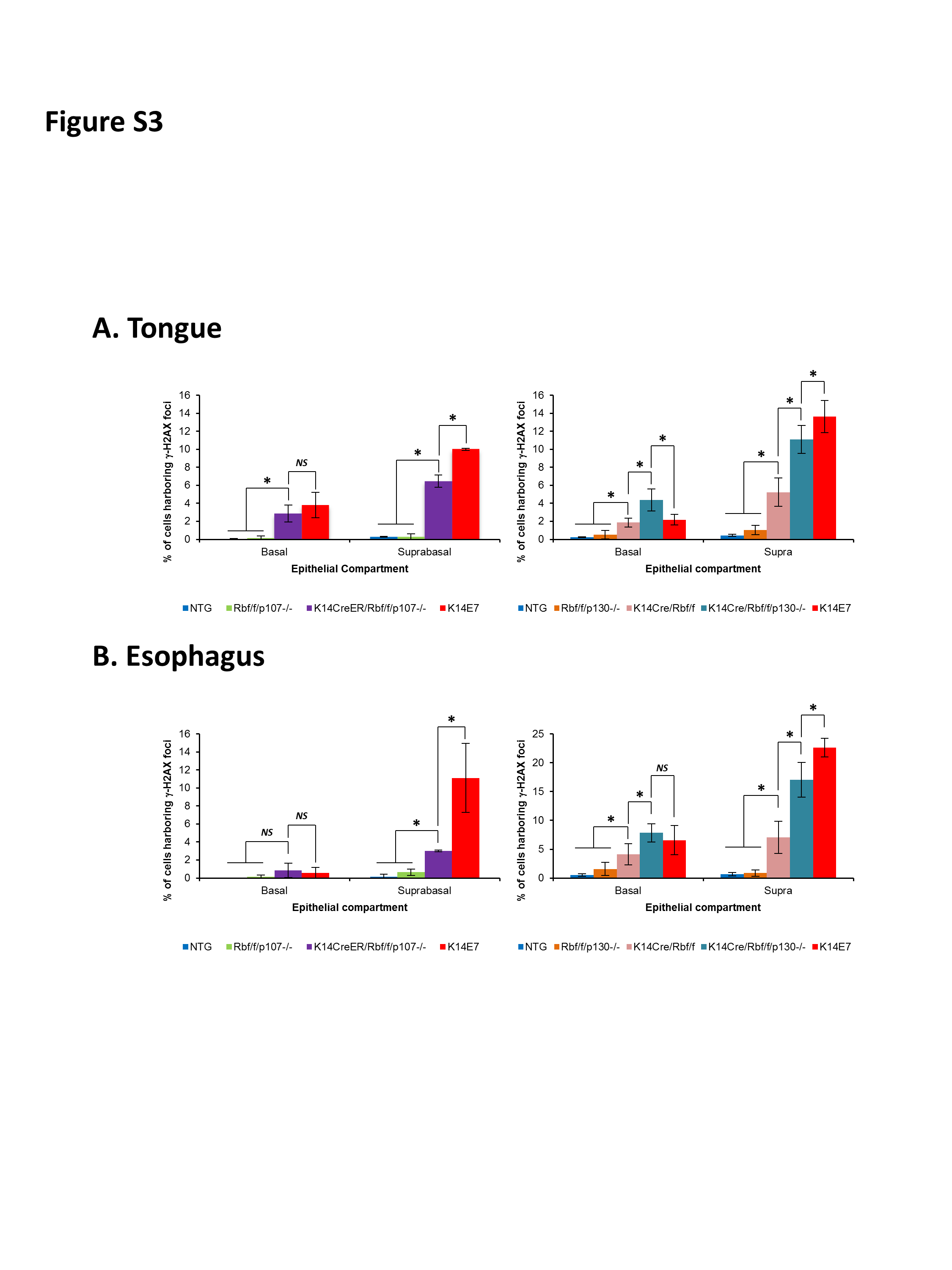

Supplement: Figure S3 — Deficiency of Pocket protein family members Increased DNA damage via γ-H2AX in the tongue and esophagus epithelia. A and B. At least three mice from each genotype were randomly selected and more than eight image frames of cells at the basal (CK14 positive) and suprabasal (CK14 negative) layers of the tongue (A) and esophagus (B) epithelia were quantified for each mouse. The amount of γ-H2AX nuclear-foci positive cells over total number of cells was plotted in each case (columns); bar, Standard deviation (SD). Asterisk (*) means significant difference (P<0.05). NS means no significant difference (P>0.05). All statistical comparisons were performed using a two-sided Wilcoxon Rank sum test. (TIF) [file pone.0075056.s003.tif]
